# *Mediator Subunit18* Controls Flowering Time and Floral Organ Identity in *Arabidopsis*

**DOI:** 10.1371/journal.pone.0053924

**Published:** 2013-01-11

**Authors:** Zhengui Zheng, Hexin Guan, Francisca Leal, Paris H. Grey, David G. Oppenheimer

**Affiliations:** 1 Department of Biology, University of Florida, Gainesville, Florida, United States of America; 2 Howard Hughes Medical Institute, Department of Molecular Genetics and Microbiology, University of Florida, Gainesville, Florida, United States of America; University of Georgia, United States of America

## Abstract

Mediator is a conserved multi-protein complex that plays an important role in regulating transcription by mediating interactions between transcriptional activator proteins and RNA polymerase II. Much evidence exists that Mediator plays a constitutive role in the transcription of all genes transcribed by RNA polymerase II. However, evidence is mounting that specific Mediator subunits may control the developmental regulation of specific subsets of RNA polymerase II-dependent genes. Although the Mediator complex has been extensively studied in yeast and mammals, only a few reports on Mediator function in flowering time control of plants, little is known about Mediator function in floral organ identity. Here we show that in *Arabidopsis thaliana*, MEDIATOR SUBUNIT 18 (MED18) affects flowering time and floral organ formation through *FLOWERING LOCUS C* (*FLC*) and *AGAMOUS* (*AG*). A *MED18* loss-of-function mutant showed a remarkable syndrome of later flowering and altered floral organ number. We show that *FLC* and *AG* mRNA levels and *AG* expression patterns are altered in the mutant. Our results support parallels between the regulation of *FLC* and *AG* and demonstrate a developmental role for Mediator in plants.

## Introduction

Mediator plays an important role in regulating RNA polymerase II (Pol II) transcription. The Mediator complex contains 22–28 subunits, and mediates interactions between transcriptional co-activators and Pol II [1], [2]. Mediator is evolutionarily conserved and has an ancient eukaryotic origin [3]; it is found in organisms from fungi to mammals and plants, although the evolutionary conservation of individual subunits is moderate [4]–[6]. More than 30 different subunits have been described that are part of the Mediator complex in different organisms, but only about 20 subunits are found in all eukaryotes [6]–[8]. The others consist of either species-specific subunits or other ancillary subunits associated with activation of specific genes. Med18 is one subunit of the Mediator complex and a component of the head module that is involved in stimulating basal RNA Pol II transcription in yeast [4], [9].

*Arabidopsis* Mediator was first found to contain 27 subunits, and most of them are conserved in eukaryotes [5]. Until now, all the known yeast/metazoan Mediator components have been identified in plants [10]. PHYTOCHROME and FLOWERING TIME1 (PFT1), now known as MED25, was identified as a factor of a Phytochrome B (phyB) signaling pathway that promotes flowering and controls final organ size [5], [11]–[14]. STRUWWELPETER (SWP)/MED14 was reported to be a nuclear protein that plays a role in defining the duration of cell proliferation [15]. Some Mediator subunits like MED25, MED8, MED16, and MED21 act as integrators in response to environmental cues in *Arabidopsis* [16]–[21]. MED18 is a subunit of the head submodule of the plant Mediator complex [10]. Recently, Kim et al. (2011) reported that several Mediator subunits including Mediator 18 (MED18) are required for microRNA biogenesis [22]. Little is known about Mediator function during floral organ formation or its role in the regulation of flowering time.

The transition from vegetative growth to reproductive development in *Arabidopsis* is regulated by multiple floral induction pathways. Genetic studies of the timing of flowering in *Arabidopsis* have revealed 5 major pathways [23]. The photoperiod and vernalization pathways integrate external signals into the floral decision, the autonomous and gibberellin (GA) pathways act independently of environmental cues, whereas the endogenous pathway adds plant age to the control of flowering time. The flowering pathways are interconnected and converge on a few floral integrators, such as *FLOWERING LOCUS T* (*FT*) and *SUPPRESSOR OF OVEREXPRESSION OF CONSTANS1* (*SOC1* or *AGL20*) [24]–[26]. One important regulator of floral initiation is the MADS-box transcription factor FLOWERING LOCUS C (FLC), which acts as a negative regulator of flowering in response to both endogenous and environmental signals; it is also an integrator of the autonomous and vernalization pathways [23], [27],[28]. The autonomous and vernalization pathways both suppress the expression of *FLC* [29], resulting in a decreased expression of *FLC* and consequently increased expression of *FT* and/or *SOC1* in a later developmental stage or after a prolonged exposure of plants to low temperature [23], [30]. The florigen, FT, directly regulates floral meristem identity genes such as *APETALA1* (*AP1*) and initiates floral morphogenesis [31], [32].

Following the vegetative to floral transition, *Arabidopsis* flowers develop four different organ types that are arranged in concentric whorls: the first whorl contains four sepals, the second whorl contains four petals, six stamens develop in the third whorl, and two fused carpels form in the fourth whorl. The control of floral organ identity has been intensively studied in the past 25 years. Analysis of floral homeotic mutants led to the proposal of a simple genetic model, explaining how three groups of regulatory genes (class A, B and C genes) alone or by interactions, control the organ identity of the four floral whorls [33], [34]. The termination of stem cells in the floral meristem requires *AGAMOUS* (*AG*), a MADS-domain transcription factor [35]; As a class C floral homeotic gene, *AG* specifies stamen identity together with the B class and *SEPALLATA* (*SEP*) genes and carpel identity together with the *SEP* genes [34], [36]. *AG* activates *SPOROCYTELESS* (*SPL*), which controls sporogenesis in both stamens and carpels [37]. Clearly, AG is one of the most important regulators for the floral transition, floral organ identity, and spore formation.

In this study, we describe the function of *Arabidopsis* Mediator subunit 18 (MED18), and show that it controls flowering time and floral organ identity by transcriptional regulation of *FLC* and *AG*.

## Materials and Methods

### Plant Materials and Growth Conditions

All the transgenic and mutant *Arabidopsis* lines used in this study were of ecotype Columbia (Col) except for the *ag-1* and *pi-1* mutants, which were in the Landsberg *erecta* (L*er*) background. Plants were grown in a temperature controlled greenhouse at 22–24°C, with a relative humidity of 60%–70%, and a16 hours light and 8 hours dark photoperiod.

For short day experiments, Plants were grown in a growth chamber equipped with 40 W fluorescent light tubes with a 9 hours light and 15 hours dark photoperiod.

### Analysis of Transcripts Levels

Semi-quantitative RT-PCR was used to measure the transcript levels of *MED18* and the floral homeotic genes using *ACTIN4* (At5g59370) and *GAPC* (AT3G04120) as loading controls. Total RNA samples were treated extensively with RNase-free DNase I to remove any contaminating genomic DNA. First-strand cDNA was synthesized using 1 µg of total RNA in a 20 µl reaction volume using High Capacity cDNA Archive Kit from Applied Biosystems (Foster City, CA) according to the manufacturer’s instructions, followed by phenol/chloroform purification, and ethanol precipitation. The cDNA were dissolved in 30 µl TE buffer and 1 µl was subjected to PCR in a 20 µl reaction volume. The RT-PCR runs were 20 to 30 cycles, depending on the linear range of PCR amplification for each gene, with cycle parameters of 94°C for 0.5 min, 58°C for 0.5 min, and 72°C for 1 min for each cycle, with a final incubation of 72°C for 10 min. *AP1*, *AP2*, *AP3*, *PI*, and *AG*, primers were designed according to published sequences [38]. All other primers designed in this study were in Table S3.

Quantitative RT-PCR was modified from a previously published method [39]. Mutant and wildtype seedling or flowers were dissected and pooled. Total RNA was extracted using RNeasy plus micro kit (Qiagen) and RNA quantity (>100 ng/µl) and purity (260/280>2.0, 260/230>1.65) were determined using a Nanodrop. RNA integrity (RIN>8.5) and 28S/18S ratio (>1.5) was assessed using a Bioanalyzer 2100 (Agilent Technologies). A quantity of 500 ng of high-quality RNA for each pooled sample was converted into cDNA using the iScript cDNA Synthesis Kit (Bio-Rad). Gene expression was determined using the CFX96TM Real Time system (Bio-Rad) with *Act2/8* [40] as a control. *FT*, *SOC1* and *FLC* Primers were designed according to Zhou and Ni [41]. The results were determined using ΔΔCt method [42], 4 replicates of pooled samples were used for both wildtype and mutant seedlings and flowers.

### *In situ* Hybridization

We used previously established methods for *in situ* hybridization [43] with the following modifications. To generate templates for *in situ* probe synthesis, a cDNA was PCR amplified using primers that contained the phage T7 RNA polymerase initiation sequence. The PCR product was used for *in vitro* transcription of digoxigenin-labeled probes using a DIG-RNA labeling kit (Roche Applied Science). DIG-labeled RNA probes were not hydrolyzed, and used at a final concentration of 400 ng/ml in the hybridization solution. Slides were photographed under bright field illumination.

### Statistical Analysis

All group differences in our dependent variables were revealed using two-tailed Student’s T-tests, and α-Levels were set at 0.05.

## Results

### *MED18* Controls Flowering Time and Floral Organ Identity

**Figure 1:**
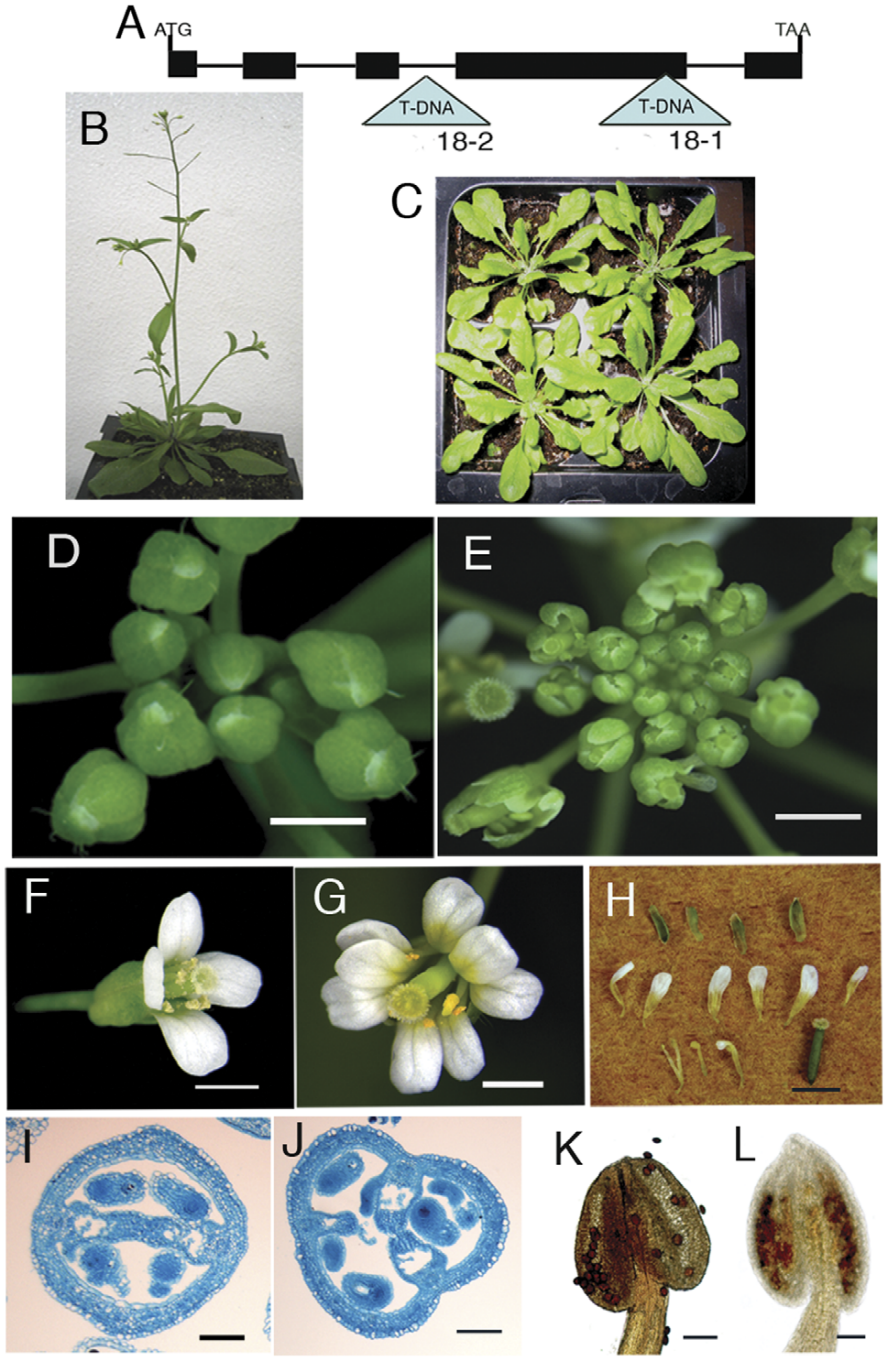
Phenotypes of *Mediator subunit 18* (*med18*) mutants. (A) Schematic diagram of the MED18 gene showing the locations of T-DNA insertions in the *med18* mutants. Black rectangles represent exons, lines represent introns, and triangles represent T-DNA insertions; the *med18-1* mutation corresponds to insertion line SAIL_889_C08, whereas the *med18-2* mutation corresponds to insertion line, SALK_ 027178. (B, C) 35 day old wild type (B), *med18-1* (C) plants. (D, E) Inflorescence of wild type (D), and *med18-1* (E) plants. (F–H) *Arabidopsis* wild type (F), *med18-1* (G), and dissected *med18-1* (H) flower. (I, J) Transverse section of wild type (I), and *med18-1* (J) carpels. (K, L) Anthers of wild type (K) and *med18-1* (L) (stained with KI/I_2_) at time of flowering. Scale bars: 1 mm in D,E,F,G and H; 100 µm in I,J,K and L.

The *MED18* (NP_565534; At2g22370) gene was originally identified through a phylogenomic comparison of single-copy genes conserved in angiosperms [44]. Additional database searches revealed that MED18 was a plant homolog of Mediator subunit 18 (Fig. S1, ref [3], [5]). To gain further insight into the function of this gene in *Arabidopsis*, we examined the phenotypes of plants homozygous for T-DNA insertions in the *MED18* coding sequence. We identified T-DNA insertion alleles (SAIL_889_C08, *med18-1*; SALK_ 027178, *med18-2*) from the SALK T-DNA insertion database (http://signal.salk.edu/cgi-bin/tdnaexpress) [45], [46], and confirmed the location of the T-DNA inserts by PCR using *MED18* and T-DNA specific primers (Fig. 1A). We also examined *MED18* mRNA levels in plants homozygous for each of the alleles using RT-PCR. *MED18* mRNA was undetectable in *med18-1* plants compared to wild type. This result suggests that *med18-1* is a null allele. Kim et al. [22] reported weak expression of *MED18* mRNA in *med18-2* plants, but we failed to detect any *MED18* mRNA using primers located on either side of the T-DNA insertion (Fig. S2).

When either *med18-1* or *med18-2* was crossed with wildtype plants, the F1 showed a wildtype phenotype, which demonstrates that both *med18-1* and *med18-2* are recessive. A complementation test was also performed using the *med18-1* and *med18-2* alleles. The F1 plants from a cross of *med18-1 and med18-2* plants showed a *med18* phenotype (Table S1), which is described below. This result demonstrates that *med18-1* and *med18-2* are allelic.

In addition to the previously described phenotype [22], *med18* mutations cause a syndrome of related phenotypes affecting flowering time, inflorescence structure, and flower morphology. Under long day conditions (16 hour light, 8 hour dark), both *med18-1* and *med18-2* mutants did not flower even after 35 days after germination (DAG) (Fig. 1C). In contrast, wildtype plants flowered approximately 14 DAG (12 leaves) (Fig. 1B). The *med18-1* mutation also affects flower morphology. In wildtype flowers, the sepals fully enclose the developing flowers until shortly after the beginning of anthesis (Fig. 1D). The sepals of *med18-1* flowers did not fully enclose the developing flowers such that the buds appeared prematurely open (Fig. 1E).

Most striking of all was that *med18-1* mutants showed dramatically altered floral organ numbers as compared to wildtype. All floral organs were affected (Table S1, Fig. 1F–H), more than 40 and 80 percent of the flowers on *med18-1* plants had abnormal sepals and petals number respectively, while >80% of the flowers had fewer than 6 stamens. Approximately 25% of *med18-1* mutant flowers had more than two carpels (Table S1, Fig. 1I, J). In addition to the altered floral organ number, *med18-1* mutants also showed delayed stamen development and later maturation of pollen (Fig. 1K, L), which led to reduce seed set.

Overexpression of *MED18* caused increased carpel and stamen numbers (Fig. S3A), and reduced petal numbers (Fig. S3B), Carpel-like sepals were also observed in *MED18* overexpressing plants (Fig. S3C), and these *MED18* overexpressing plants flowered earlier than wildtype (data not shown).

### *MED18* Expression during *Arabidopsis* Development

**Figure 2:**
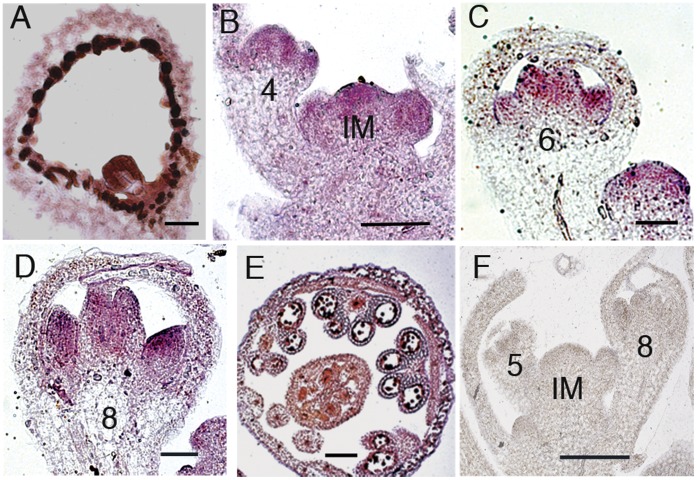
Expression patterns of *MED18*. (**A–D**) Longitudinal sections of wild type show *MED18* mRNA detected in (A) developing seed, (B) inflorescence meristem, floral meristem and sepals, (C, D) petal, stamen, and carpel primordia. (**E**) Transverse section of a wild type flower shows *MED18* mRNA expression in all floral organs. (**F**) *MED18* mRNA sense strand control showing no non-specific hybridization. Numbers in B–D and F indicate flower stages; IM in B and F indicates inflorescence meristem. Scale bars: 100 µm in B–F and 25 µm in A.

The flowering time and floral organ patterning defects in the *med18* mutants suggests that *MED18* plays a role in regulating genes important for flowering and floral organ development. To determine if *MED18* expression coincides with the phenotype, we examined the RNA expression pattern of *MED18* in wildtype plants using *in situ* hybridization. *MED18* transcripts could be detected in developing seeds (Fig. 2A), the inflorescence meristem, the floral meristem, and floral organ primordia (Fig. 2 B and C). Transcript abundance appeared highest in the developing stamens and pistils (Fig. 2D). In later stages, *MED18* expression was abundant in developing ovules and pollen (Fig. 2E) and in addition, *MED18* mRNA was weakly expressed in petals, sepals, and the walls of carpels up to stage 12 (Fig. 2E). The strong expression of *MED18* in the inflorescence meristem and floral organs is consistent with the floral defects observed in *med18-1* mutants.

### *med18* Mutants Affect Flowering Time in Both Long and Short Days

**Figure 3:**
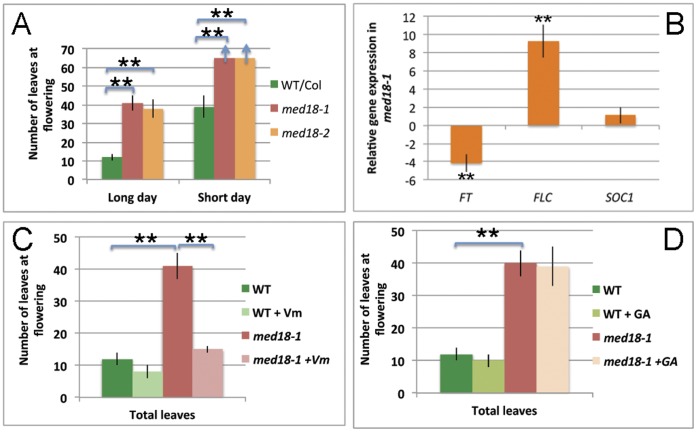
*MED18* controls flowering time. (**A**) *MED18* controls flowering time under both long day and short day conditions. Arrow indicates that flowering was not achieved when the experiment was terminated. (**B**) Transcripts levels of *FT* and *FLC* are altered in *med18* mutants. (**C, D**) Flowering time in vernalization (C) and gibberellin (D) treatment. Vm: vernalization, GA: gibberellin. Scale bars indicate mean ± s.e.; **p≤0.01.

In long day condition (16 h light, 8 h dark), the mutant plants did not flower until more than 40 leaves; and in short day (9 h light, 15 h dark), after 95 days (more than 60 leaves), *med18* mutants still did not flower, and many rosette and cauline leaves showed senescence (Fig. 3A). Compared to wildtype plants (12 leaves under long days and about 40 leaves under short days), *med18* is late flowering under both long and short days (Fig. 3A).

### *med18* Mutants Affect Flowering Time through up-regulation of *FLC* Expression

To understand how MED18 regulates flowering time, we compared the expression levels of key genes of different pathways in mutant and wildtype seedlings using RT-PCR. *FT* and *FLC* show expression differences in the *med18* mutants as compared to wildtype plants (Fig. S4). We performed QPCR and showed that *FT* mRNA was down regulated approximately 4.7 fold (Fig. 3B, p≤0.004), while the *FLC* transcript level was much higher (9.5 fold) in *med18-1* than in the wildtype plants (Fig. 3B, p≤0.001). For *FT* and *SOC1*, which are downstream of *FLC*, only *FT* mRNA was significantly suppressed by the loss of *MED18* function, whereas *SOC1* mRNA levels show no significant difference (Fig. 3B, p = 0.106).

### *med18* Mutants are Responsive to Vernalization but not GA Treatment

To examine the responsiveness of *med18* to vernalization, the *med18-1* plants were planted and kept at 4°C for 4 weeks in the dark and then transferred to normal growth temperature (23°C). The vernalization-treated *med18* plants flowered much earlier than the untreated plants, after producing about14 leaves, which is comparable to that of wildtype plants (Fig. 3C. The untreated plants flowered after producing close to 40 leaves, which suggests that the flowering of the mutant plants was decreased by vernalization (Fig. 3C). To examine the effects of GA treatment, a GA solution of 20 µM was sprayed twice a week after germination until flowering. The results show that GA has no obvious effect on the flowering of *med18* mutants as compared to wildtype plants. The GA-treated *med18-1* plants initiated flowering after producing more than 35 leaves, which is not significantly different than untreated mutant plants (p = 0.115, Fig. 3D). Our results are consistent with previous results that show that vernalization promotes flowering by repressing *FLC* and releasing *FT* from repression [23], [47]. The responsiveness of *med18-1* to vernalization suggests that *MED18* regulates flowering time through the vernalization pathway.

### *MED18* Affects Floral Organ Formation in all Four Whorls

**Figure 4:**
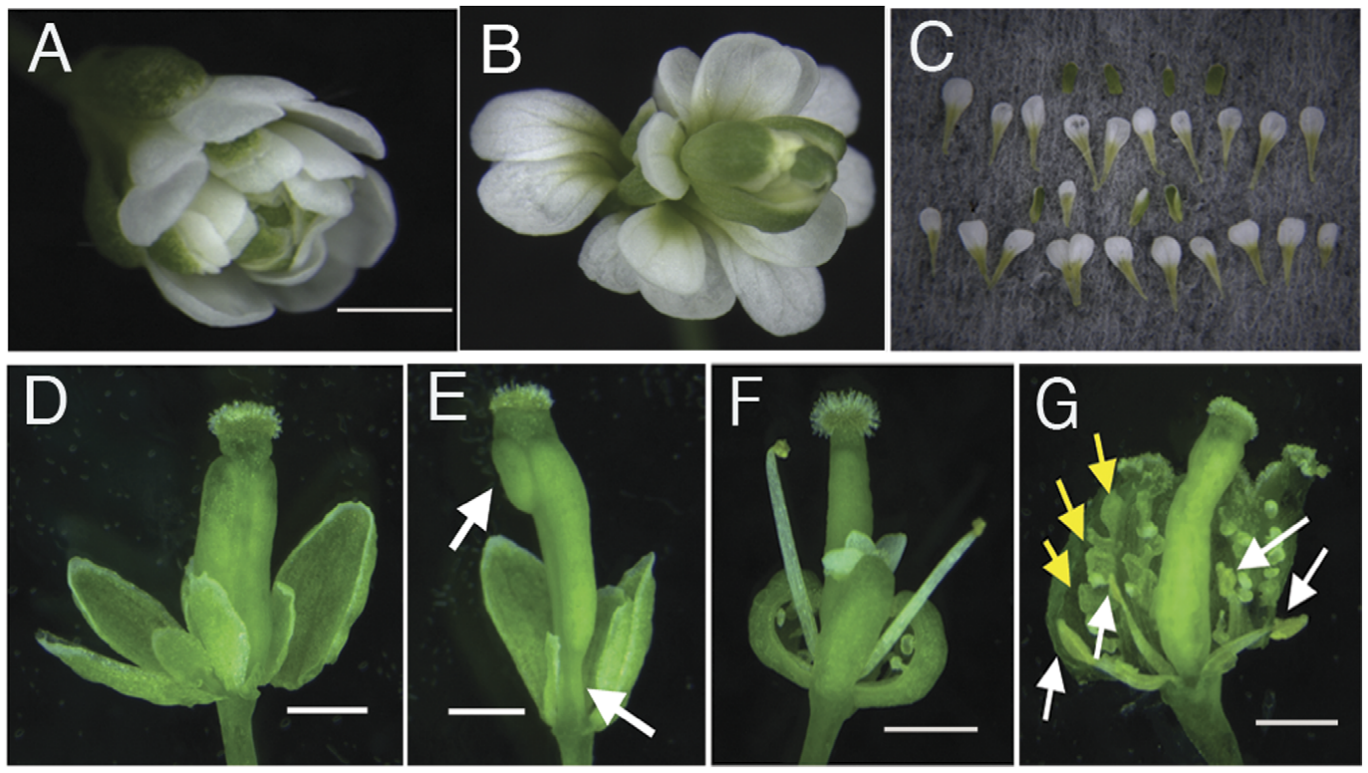
Double mutant of *med18-1* and floral homeotic genes. (**A**) *ag-1* flower. (**B**) *med18-1 ag-1* double mutant flower. (**C**) dissection of *med18-1 ag-1* double mutant flower. (**D**) *pi-1* flower. (**E**) *med18-1 pi-1* double mutant flower. (**F**) *ap2-5* flower. (**G**) *med18-1 ap2-5* double mutant flower. Scale bars: 1 mm.

Both mutation and overexpression of *MED18* altered the number of floral organs (Fig. 1G–I, Table S1, Fig. S3). The increased number of petals and fewer than normal stamens seen in the *med18* mutants was reminiscent of the floral phenotype of *agamous* (*ag*) mutants [48], [49]. To further explore the floral developmental pathway in which *MED18* is involved, we constructed double mutants with the well-studied floral developmental regulators, *ag*, *pistillata* (*pi*) and *apetala2* (*ap2*). Flowers on the *med18-1 ag-1* double mutant showed the striking reiteration of sepals and petals characteristic of *ag* mutants (Fig. 4A–C, Table S2). The *med18-1 pi-1* double mutant flowers had abnormal carpels, but fewer sepals than the *pi-1* single mutant (Fig. 4D, E, Table S2). The flowers on the *med18-1 ap2-5* double mutant displayed a much more complex phenotype. Carpels in both the first and fourth whorls were present as seen in the *ap2-5* single mutant. In addition, double mutant flowers exhibited petaloid stamens and other petaloid structures in whorls 2 and 3 (Fig. 4F, G, Table S2).

### *MED18* Affects Floral Organ Formation through Regulation of Floral Homeotic Gene Expression

**Figure 5:**
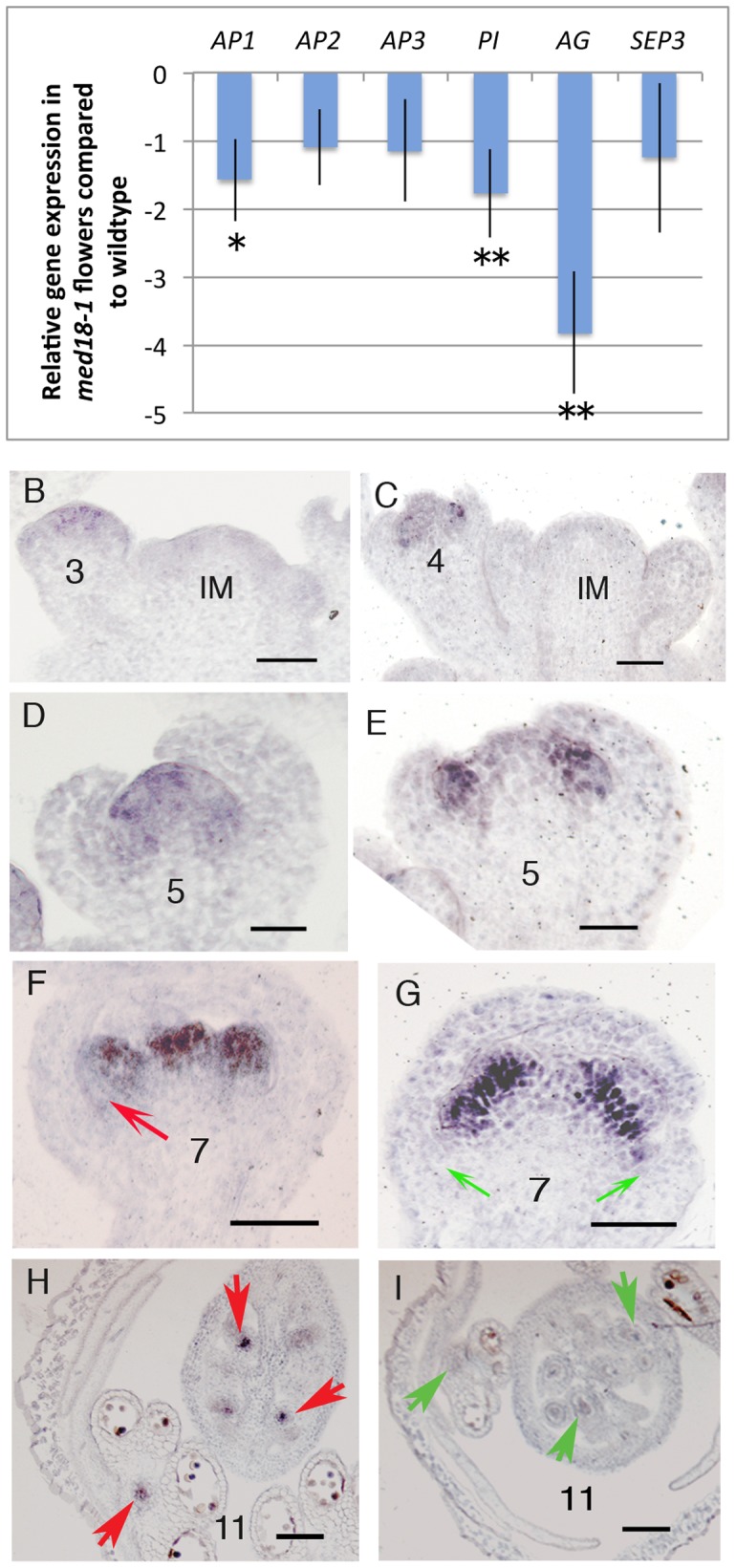
MED18 regulates floral homeotic gene expression. (A) Relative transcript levels of six floral homeotic genes determined by real-time RT-PCR in wildtype and *med18-1* mutants (**p≤0.01, *p≤0.05). (B, D, F, H) *AG* expression pattern as determined by *in situ* hybridization in wild type flowers. (C, E, G, I) The *AG* expression pattern in *med18-1* mutant flowers. Numbers in B–I show flower stages, IM in B and C indicates inflorescence meristem. Arrows in (H) and (I) indicate expression in vascular bundles of stamens and carpels, in (F) and (G) showing developing pedals. Scale bars in B–I: 50 µm.

The clear epistasis observed in the *med18-1 ag-1* double mutants suggested that *MED18* and *AG* function in the same pathway to control floral development. Therefore, we examined *AG* mRNA levels as well as the levels of mRNA for other key floral regulators in *med18* mutants using semi-quantitative RT-PCR. Our results showed that the mRNA expression levels of *AG*, *AP1* and *PI* were down regulated in *med18-1* mutants while *AP2* and *AP3* mRNA showed no obvious change (Fig. S5). QPCR results revealed *AG* mRNA was down regulated up to 4.8 fold (Fig. 5A, p≤0.007), *AP1* and *PI* mRNA expression levels were also reduced in the *med18-1* mutant 1.9 (p≤0.014) and 2.4 fold (p≤0.011) respectively, while *AP2* and *AP3* transcript levels showed no significant differences between *med18-1* and the wildtype (Fig. 5A, p = 0.068 and 0.082). The decreased *AG* expression is likely to cause a stamen to petal transition as well as abnormal carpel development [49], as observed in *med18-1 and MED18* overexpression flowers (Fig. 1F–H, Fig. S3). These results support the hypothesis that *MED18* controls floral organ identity through its regulation of floral homeotic gene expression.

### Altered *AG* Expression Patterns are Observed in *med18* Mutants

To further examine the expression of *AG* in *med18-1* mutants, *in situ* hybridization using an *AG* antisense RNA probe was performed on tissue sections from wildtype (Fig. 5B, D, F, H) and *med18-1* plants (Fig. 5C, E, G, I). As described previously [50], [51], *AG* is not expressed in the inflorescence meristem, nor in stage 1 or stage 2 floral meristems in wildtype plants. Strong *AG* expression is first found in the center of stage 3 and stage 4 wildtype flowers, but not in the emerging sepal primordia (Fig. 5B). During later stages of wildtype flower development, *AG* expression is present in stamens and carpels (Fig. 5D, F, H). In *med18-1* mutants, a pattern of *AG* expression similar to that seen in wildtype was observed in both the inflorescence meristem and in stage 1 and stage 2 floral meristems (Fig. 5C). In flowers from stage 4 to stage 7, weak *AG* expression was detected in the center of the carpel primordia, but strong *AG* expression was observed in stamen primordia, although no expression was observed in petal and sepal primordia (Fig. 5C, E, G). In later development stages (stage 11, Fig. 5H, I), *AG* expression was observed in developing pollen similar to that observed in wildtype flowers (Fig. 5H), but it was difficult to detect any signal in vascular bundles of stamens and carpels (Fig. 5I). This result, together with the results of the QPCR analysis, strongly suggests that *MED18* is required to maintain the normal *AG* expression level and pattern during early stamen and gynoecium development.

## Discussion

Under many conditions Mediator appears to function as a general transcription factor [52]. Nonetheless, expression profiling of yeast Mediator subunit mutants has revealed the direct regulation of specific sets of genes by Mediator [53], and analysis of Mediator in *Arabidopsis* has shown that the Mediator subunits are important in regulating specific developmental processes like early embryo patterning [54], [55], cell number and organ size [12], [15], flowering time control [5], [14], [56] environmental regulation and defense gene regulation [16], [18]–[21], [57].

**Figure 6:**
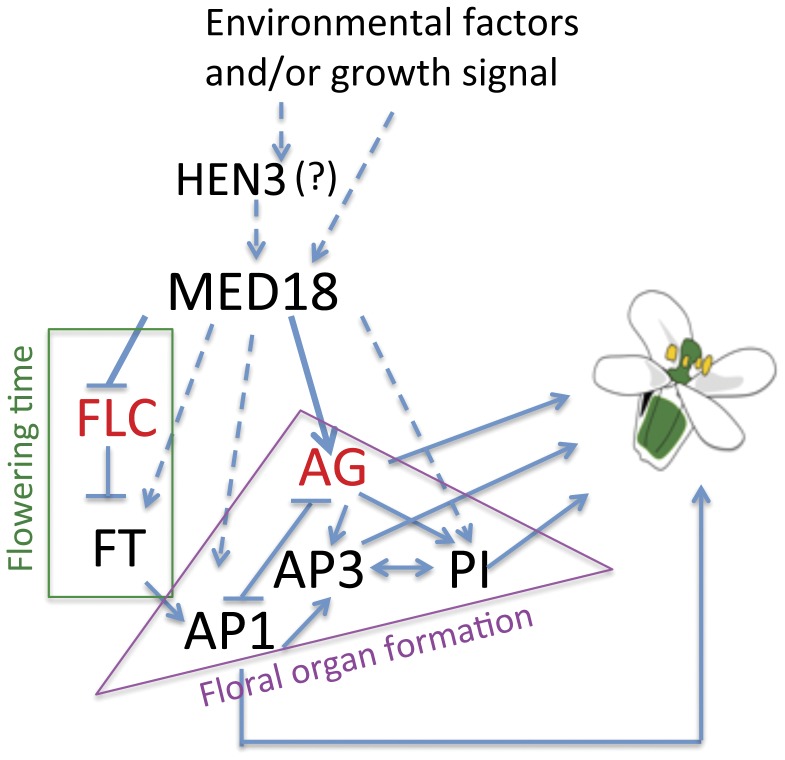
Model of *MED18* control flowering time and floral organ identity. Growth signals are transmitted to the Mediator complex by direct action or through HEN3 on MED18 to regulate the transcription of target genes. Both flowering time integrator FLC and floral organ identity organizer AG transcripts levels are determined by MED18. In addition, MED18 affects the transcription of other target genes, which are flowering time and floral organ identity regulators, such as *FT*, *AP1* and *PI*. These regulators work together and with other factors to control flowering and floral organ formation.

MED18 was first characterized as a general transcription factor that promotes Pol II transcription through promotion of the transcription of miRNA, and knocking down *MED18* expression caused abnormal cotyledon and silique development as well as a later flowering phenotype [22]. However, the mechanisms by which MED18 regulates flowering are poorly understood, and nothing is known about how MED18 regulates floral organ identity. In this study, we identified MED18 as a regulator of both flowering time and floral organ identity. Our findings show that MED18 controls flowering time by up-regulating *FLC* expression, which also affects the expression of the downstream gene, *FT*. After the flowering transition, MED18 plays a role in floral organ identity by regulating the *AG* expression level and pattern as well as *AP1* expression levels (Fig. 5, 6). These results suggest that MED18 is important in the integration of key signaling pathways in plants by controlling target genes transcription (Fig. 6).

The *med18* mutants display various developmental defects, suggesting that MED18 regulates multiple pathways. This is in agreement with the function of animal and yeast Mediator proteins, which have been suggested to regulate both basic and specific transcription [8], [58], [59]. We have focused on a study of MED18 function during flowering and floral organ development. We found that *MED18* mRNA is localized mainly to the precursor cells of inflorescence meristem, the four floral whorls and strongly in the pollen and ovule primordia. This suggests a role for MED18 in the control of floral and reproductive organ initiation and development. Floral primordia arise from the inflorescence meristem, and floral organs are then formed in the floral meristem. The ABC model of flower development explains how three classes of genes control sepal, petal, stamen, and carpel identity [33]. Furthermore, the model indicates that class A and C genes are mutually antagonistic [60]. The previously isolated floral homeotic genes all seem to code for potential transcription factors. AP1, AP3, PI, AG, and the SEP proteins contain the MADS domain known to bind to DNA [36]. AP2 contains another DNA binding domain, the AP2 domain [61]. General regulators like Mediator are likely to regulate these genes. Our studies have shown that mutations in *MED18* cause down regulation of several homeotic genes, such as *AP1*, *PI* and *AG*, but not all of them; this finding suggests that the effect of MED18 on homeotic gene expression is gene specific.

Because MED18 affects the expression of multiple genes, the flowers of *med18* mutants show variable phenotypes, such as 4–14 petals, 2–6 stamens, 0–6 sepals and 1–3 carpels. In one of the flowers that had 14 petals, four stamens are clearly visible (Fig. S6). Therefore, the effect of the *med18* mutation cannot be a simple homeotic transformation of the floral organs into petals. It is interesting to note that the *med18* mutation causes a down regulation of *AG* expression, yet some flowers have three carpels. According to the ABC model, down regulation of an A class gene (such as AP1) will cause ectopic carpel formation.

The later flowering phenotype observed in *med18* mutants is caused by up-regulation of *FLC* mRNA expression. FLC also contains a MADS domain, and interestingly, both *FLC* and *AG* expression levels are altered in *med18* mutants, but in opposite directions. There are similarities between the DNA structure of *FLC* and *AG*. Most of the genes in the *Arabidopsis thaliana* genome have short (<1 kb) introns [62], but intron 1 in *FLC* and intron 2 in *AG* are both greater than 3.0 kb in length, and both of these introns have cis-elements that are important for transcriptional control [27], [63], [64]. *HUA1*, *HUA2*, *HUA ENHANCER2* [*HEN2*] and *HEN4* were shown to positively regulate *AG* expression, either by inhibiting premature polyadenylation within *AG* intron 2 or by promoting the splicing of this intron, and *hua1 hua2* double mutants have reduced levels of *FLC* mRNA [65]. All these results and our data that *MED18* regulates *FLC* and *AG* mRNA expression levels, strongly support the idea of parallel regulation of *FLC* and *AG* [66].

The result of up-regulation of *FLC* and down-regulation of *AG* suggest that *MED18* is not a general transcription repressor, but rather it plays different roles depending on the identity of its target, and/or the developmental stage. The mechanism by which MED18 affects *FLC* and *AG* expression is currently unknown. Highly specific gene regulation is thought to be determined by activators and combinatorial use of cofactors. In yeast, Med18 acts downstream of CDK8, and may act as a direct processor of signaling pathways for determining specific gene expression [53]. Med18 was also reported to be required for proper termination of transcription of a subset of genes during yeast budding [67]. In *Arabidopsis*, the CDK8 homolog is *HUA ENHANCER3* [*HEN3*], which also controls organ identity and show similar loss-of-C-function phenotypes (Wang and Chen, 2004), suggesting that *HEN3* may regulate organ identity through *MED18* in *Arabidopsis* (Fig. 6). Our results suggest a conserved mechanism may exist in yeast and plants.

## Supporting Information

Figure S1 **Phylogenetic tree of eukaryotic** ***MEDIATOR SUBUNIT 18*** **(** ***MED18*** **).** The Bayesian inference analysis was derived from 406 amino acid positions of MED18 in different species of Eukaryotes. The best model amino acid replacement for MED18 sequences was JTT and gamma model for substitution rate heterogeneity between sites. Bayesian phylogenetic inference was performed with MrBayes Version 3.0 using four chains and 2,000,000 generations. Numbers at node indicate posterior probabilities; scale bar shows 0.2 amino acid substitutions per site.
(TIF)

Figure S2 **RT-PCR result of** ***MED18*** **showing that** ***MED18*** **mRNA was not detected in** ***med18-1*** **,** ***med18-2*** **, and** ***med18-1/med18-2*** **T1 plants using primers designed for the PCR product including both insertion sites.** The *GAPC* gene was used as a control.
(TIF)

Figure S3 **Phenotype of over expression of** ***MED18*** **in transgenic plants transformed with a** ***35S:: MED18*** **construct.**
(TIF)

Figure S4 **RT-PCR results of selected flowering time regulators in wild type and** ***med18-1*** **seedlings.** In *med18-1* plants, *FLC* is up-regulated, *FT* is down-regulated, but others show no obvious difference.
(TIF)

Figure S5 **RT-PCR results of selected floral organ identity genes in wild type and** ***med18-1*** **flowers.** In *med18-1* plants, *AP2*, *PI* and *AG* are down-regulated, but others show no obvious difference.
(TIF)

Figure S6 **A** ***med18-1*** **mutant flower that shows 14 petals and 4 stamens.**
(TIF)

Table S1 **Number of different organ types in** ***med18*** **flowers.^a^** a 200 flowers were counted. b mean of 200 lowers. c Organ number is 6 or more. d Organ number is 4 or less. e Organ number is 1 or 1.5 (some carpels only have half or one third of normal size located at the tip of pistil ). f Organ number is between 2 and 3 (same as e). Mutant plants were grown in the greenhouse (16 h light, 23±2°C), wildtype plants were grown under the same conditions. The floral organs on 200 wildtype plants were also counted, and all flowers showed 4 sepals, 4 petals, 6 stamens and 2 fused carpels, except 2 flowers showed 5 petals. The *med18-1* allele is a strong allele and all four floral organs show significant differences from wildtype (**p<0.01), *med18-2* is a weaker allele, and only petals and stamens show significant differences from wildtype. The F1 plants from a cross of *med18-1* with *med18-2* (*med18-1×med18-2*) also show obvious floral organ number changes.
(DOCX)

Table S2 **Number of different organ types in floral homeotic mutants and double mutants with** ***med18-1*** **flowers.^a^** a, at least 15 flowers were counted for each mutant. b, the first whorl of each organ showed the normal organ number for that whorl. c, many flowers showed 1 connected carpel-like sepal.
(DOCX)

Table S3 **Primers designed in this study.**
(DOCX)
