# Supplementary figures and images for: Mediator Subunit18 Controls Flowering Time and Floral Organ Identity in Arabidopsis
Source: PLoS One. 2013 Jan 11;8(1):e53924. doi: 10.1371/journal.pone.0053924 (PMC3543355; doi:10.1371/journal.pone.0053924)

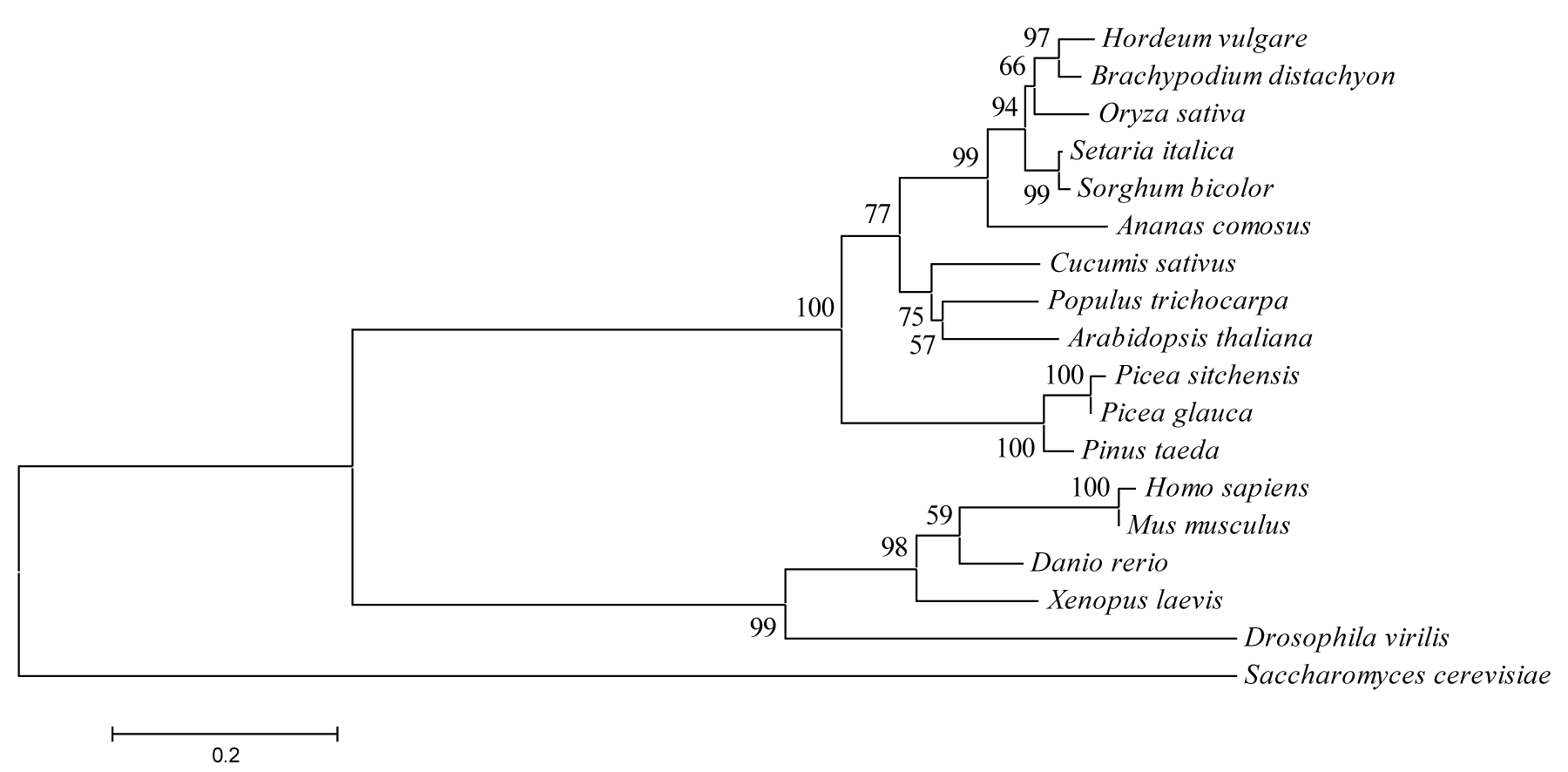

Supplement: Figure S1 — Phylogenetic tree of eukaryotic MEDIATOR SUBUNIT 18 ( MED18 ). The Bayesian inference analysis was derived from 406 amino acid positions of MED18 in different species of Eukaryotes. The best model amino acid replacement for MED18 sequences was JTT and gamma model for substitution rate heterogeneity between sites. Bayesian phylogenetic inference was performed with MrBayes Version 3.0 using four chains and 2,000,000 generations. Numbers at node indicate posterior probabilities; scale bar shows 0.2 amino acid substitutions per site. (TIF) [file pone.0053924.s001.tif]

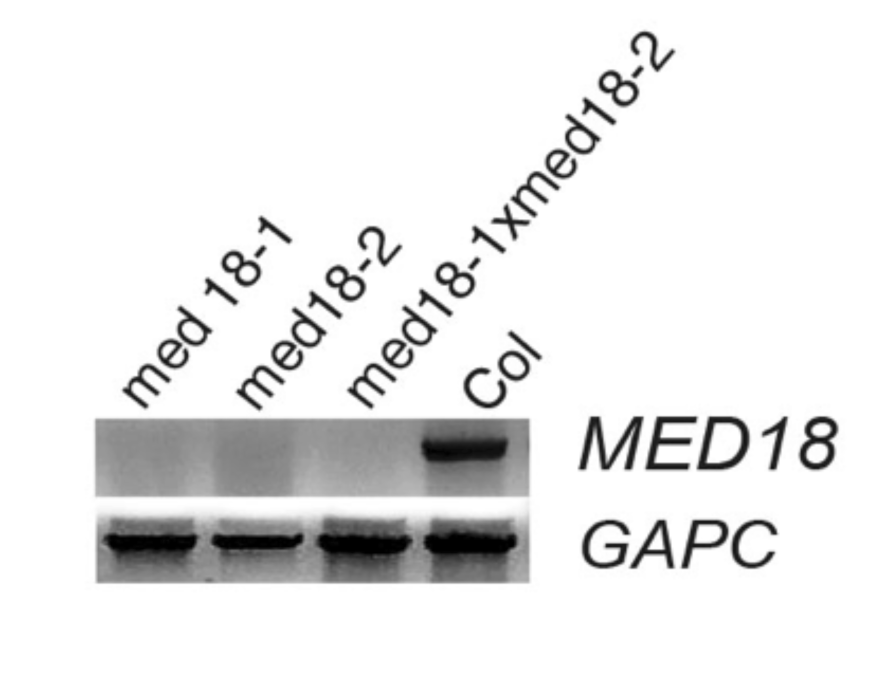

Supplement: Figure S2 — RT-PCR result of MED18 showing that MED18 mRNA was not detected in med18-1 , med18-2 , and med18-1/med18-2 T1 plants using primers designed for the PCR product including both insertion sites. The GAPC gene was used as a control. (TIF) [file pone.0053924.s002.tif]

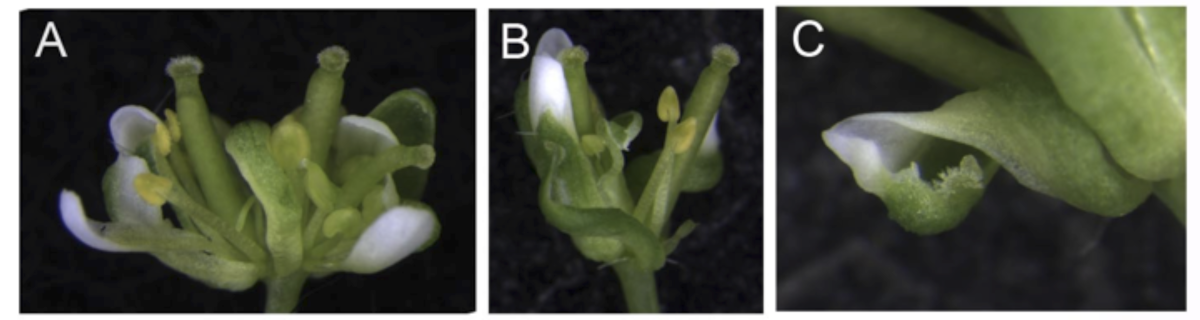

Supplement: Figure S3 — Phenotype of over expression of MED18 in transgenic plants transformed with a 35S:: MED18 construct. (TIF) [file pone.0053924.s003.tif]

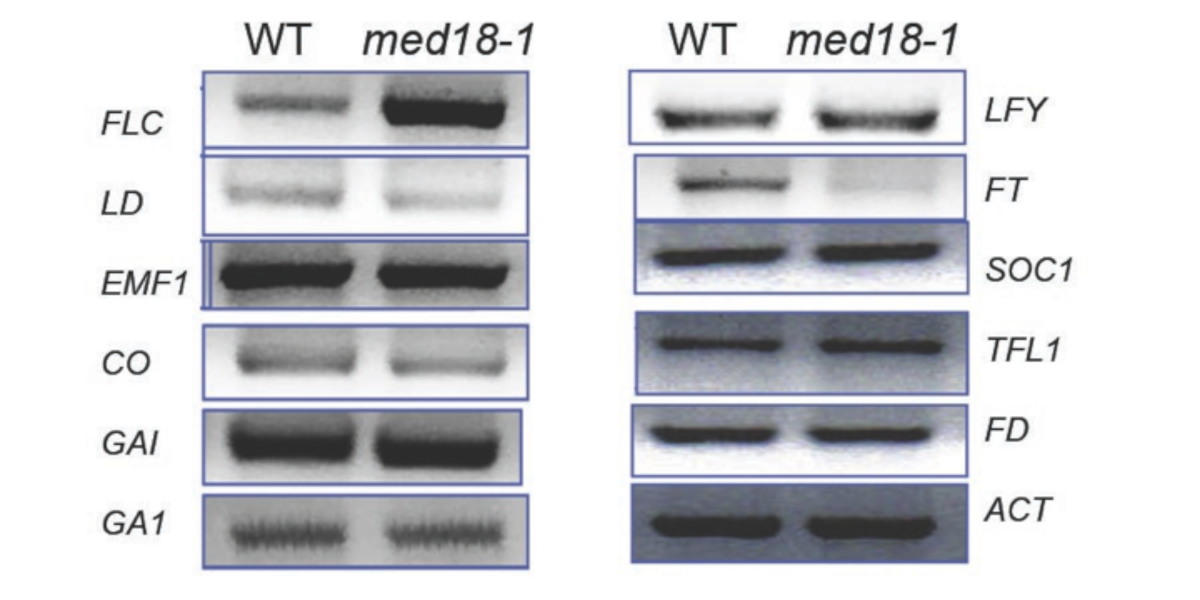

Supplement: Figure S4 — RT-PCR results of selected flowering time regulators in wild type and med18-1 seedlings. In med18-1 plants, FLC is up-regulated, FT is down-regulated, but others show no obvious difference. (TIF) [file pone.0053924.s004.tif]

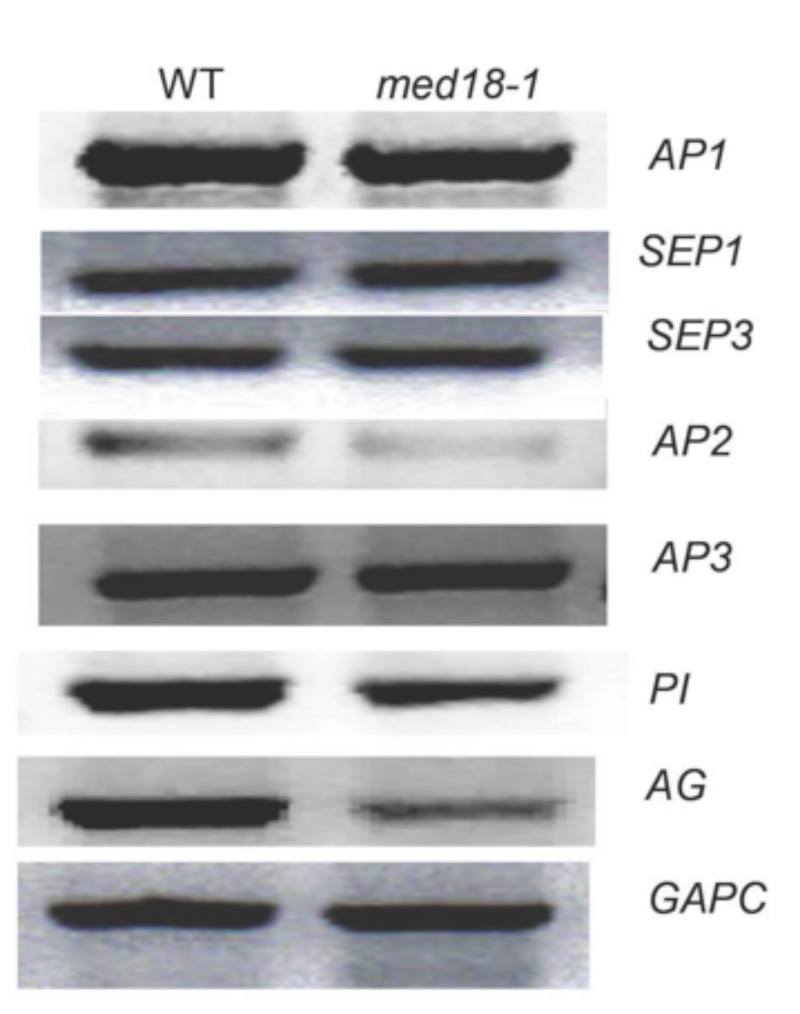

Supplement: Figure S5 — RT-PCR results of selected floral organ identity genes in wild type and med18-1 flowers. In med18-1 plants, AP2, PI and AG are down-regulated, but others show no obvious difference. (TIF) [file pone.0053924.s005.tif]

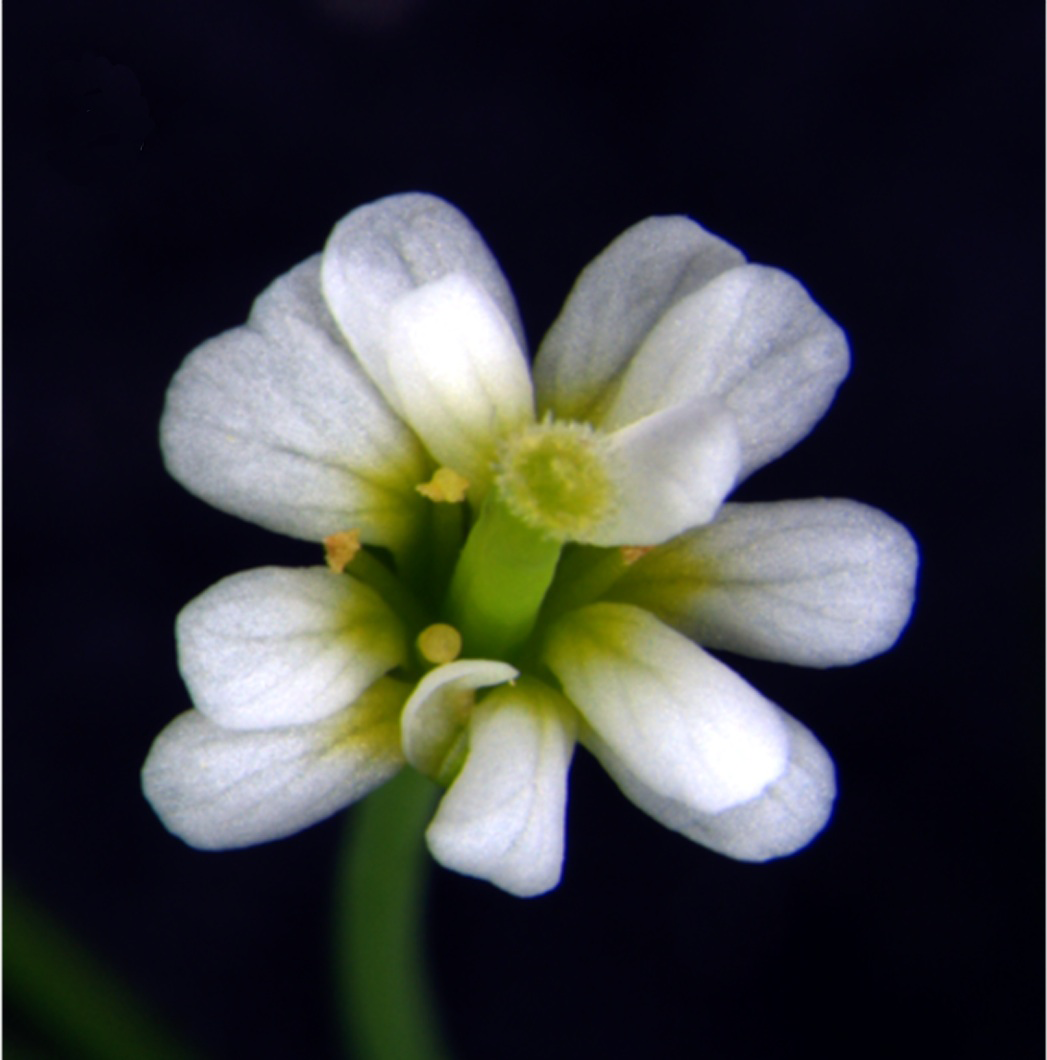

Supplement: Figure S6 — A med18-1 mutant flower that shows 14 petals and 4 stamens. (TIF) [file pone.0053924.s006.tif]
