# Supplementary material for: Mediator Subunit18 Controls Flowering Time and Floral Organ Identity in Arabidopsis
Source: PLoS One. 2013 Jan 11;8(1):e53924. doi: 10.1371/journal.pone.0053924 (PMC3543355; doi:10.1371/journal.pone.0053924)
Supplement: Table S1 — Number of different organ types in med18 flowers.a a 200 flowers were counted. b mean of 200 lowers. c Organ number is 6 or more. d Organ number is 4 or less. e Organ number is 1 or 1.5 (some carpels only have half or one third of normal size located at the tip of pistil ). f Organ number is between 2 and 3 (same as e). Mutant plants were grown in the greenhouse (16 h light, 23±2°C), wildtype plants were grown under the same conditions. The floral organs on 200 wildtype plants were also counted, and all flowers showed 4 sepals, 4 petals, 6 stamens and 2 fused carpels, except 2 flowers showed 5 petals. The med18-1 allele is a strong allele and all four floral organs show significant differences from wildtype (**p<0.01), med18-2 is a weaker allele, and only petals and stamens show significant differences from wildtype. The F1 plants from a cross of med18-1 with med18-2 (med18-1×med18-2) also show obvious floral organ number changes. (DOCX) [file pone.0053924.s007.docx]

| Organ type | Organ number | *med18-1* | | *med18-2* | | *med18-1× med18-2* | |
| --- | --- | --- | --- | --- | --- | --- | --- |
|  |  | (%) | Average number ^b^ | (%) | Average number ^b^ | (%) | Average number ^b^ |
| Sepal | 3 | 14 | 4.34** | 4 | 4.03 | 5 | 4.26** |
|  | 4 | 56 |  | 87 |  | 74 |  |
|  | 5 | 21 |  | 7 |  | 15 |  |
|  | ^c^ 6+ | 9 |  | 2 |  | 6 |  |
| Petal | 4 | 16 | 5.47** | 84 | 4.34** | 48 | 5.20** |
|  | 5 | 21 |  | 10 |  | 11 |  |
|  | ^c^ 6+ | 63 |  | 6 |  | 39 |  |
| Stamen | ^d^ 4- | 58 | 4.59** | 4.5 | 5.73** | 41 | 4.97** |
|  | 5 | 25 |  | 18 |  | 21.5 |  |
|  | 6 | 17 |  | 77.5 |  | 37.5 |  |
| Carpel | ^e^ 1 | 8.5 | 2.24** | 2 | 1.98 | 4.5 | 2.03 |
|  | 2 | 59 |  | 98 |  | 88.5 |  |
|  | ^f^ 3 | 32.5 |  | 0 |  | 7 |  |
