# Supplementary material for: Mediator Subunit18 Controls Flowering Time and Floral Organ Identity in Arabidopsis
Source: PLoS One. 2013 Jan 11;8(1):e53924. doi: 10.1371/journal.pone.0053924 (PMC3543355; doi:10.1371/journal.pone.0053924)
Supplement: Table S2 — Number of different organ types in floral homeotic mutants and double mutants with med18-1 flowers.a a, at least 15 flowers were counted for each mutant. b, the first whorl of each organ showed the normal organ number for that whorl. c, many flowers showed 1 connected carpel-like sepal. (DOCX) [file pone.0053924.s008.docx]

| mutant  organ type | Intermediate  organ | *ap2-5* | *med18-1*  *ap2-5* | *Pi-1* | *med18-1*  *Pi-1* | *ag-1* | *med18-1 ag-1* |
| --- | --- | --- | --- | --- | --- | --- | --- |
| Sepal | Sepal ^b^ | 0.4 | 0.2 | 4.6 | 3.9 | 4.2 | 3.6 |
|  | Petal like | 1.7 | 0 | 4.2 | 5.3 |  |  |
|  | Stamen like | 0 | 0 | 0 | 0 |  |  |
|  | Carpel like | 3.7 | 1.8 ^c^ | 0 | 0 |  |  |
| Petal | Petal ^b^ | 2.6 | 1.2 | 0.3 | 05 | 10.1 | 10.3 |
|  | Sepal like | 1.7 | 0 | 4.2 | 5.3 |  |  |
|  | Stamen like | 2.5 | 6.7 | 2.6 | 2.8 |  |  |
|  | Carpel like | 1.3 | 0.6 | 0 | 0 |  |  |
| Stamen | Stamen ^b^ | 5.6 | 2.3 | 0.5 | 0.1 | 0 | 0 |
|  | Sepal like | 0 | 0 | 0 | 0 |  |  |
|  | Petal like | 2.5 | 6.7 | 2.6 | 2.8 |  |  |
|  | Carpel like | 0 | 0 | 0 | 0 |  |  |
| Carpel | Carpel ^b^ | 2 | 1.6 | 2.7 | 1.6 | 0 | 0 |
|  | Sepal like | 3.7 | 1.8 ^c^ | 0 | 0 |  |  |
|  | Petal like | 1.3 | 0.6 | 0 | 0 |  |  |
|  | Stamen like | 0 | 0 | 0 | 0 |  |  |
