# Supplementary material for: Mediator Subunit18 Controls Flowering Time and Floral Organ Identity in Arabidopsis
Source: PLoS One. 2013 Jan 11;8(1):e53924. doi: 10.1371/journal.pone.0053924 (PMC3543355; doi:10.1371/journal.pone.0053924)
Supplement: Table S3 — Primers designed in this study. (DOCX) [file pone.0053924.s009.docx]

| Gene name | Sequence |
| --- | --- |
| **Arabidopsis mutant confirmation primers** | |
| Left-P | ATTCGCTCCCGAGGACGATTTA |
| Right-P | ACGGTTTCGACAAGTGAACAAGCC |
| ***MED18* overexpression primers** | |
| For | CTCGAGTTCGCGTTATTAGCACGACGCT |
| Rev | CTGCAGAGGTTGAGAGAAGAGCAAAGATCA |
| ***MED18* gene RNA probe template primers** | |
| For | ACATGTTGGTGGTGCGATGAGA |
| Rev | AGATGAAACAGCAGCAGCGACT |
| T7 added | TAATACGACTCACTATAGGG |
| **RT-PCR primers** | |
| *MED18* For | CGAACCCACATGGACGGTTAAA |
| *MED18* Rev | AGATGAAACAGCAGCAGCGACT |
| *TFL1* For | CACTTTGGTGATGATAGACCCAGA |
| *TFL1* Rev | TTGCGTGCAGCGGTTTCTCTTT |
| *CO* For | TCCATTAACCATAACGCATACATTTCATCC |
| *CO* Rev | TTCTCTCTTTGCGAACCGGCCAT |
| *SOC1* For | ACTCTTGGGAGAAGGCATAGGA |
| *SOC1* Rev | TGGGCTACTCTCTTCATCACCT |
| *FD* For | GCGCTGGCTTCGAGTTTCT |
| *FD* Rev | GCATTCCTGTTTCCTAGCG |
| *GA1* For | TCGGCTTACGATACAGCTTGGGTT |
| *GA1* Rev | TGGGAACTCCTCCATTGAAACG |
| *GAI* For | AACTCGGCATGTTGTCCTGGTT |
| *GAI* Rev | AGCCAGCTTACACCCAACTTCA |
| *LD* For | GCTTCTGTGCTGATCTGGTTT |
| *LD* Rev | CAAGCATGTGTTTCTTGGTGGA |
| *LFY* For | TTGATGCTCTCTCCCAAGAAGGGT |
| *LFY* Rev | TTTCGCCACGGTCTTTAGCA |
| *FLC* For | TCACCTTCTCCAAACGTCGCAA |
| *FLC* Rev | TGAGTTCGGTCTTCTTGGCTCT |
| *EMF1* For | CATGCGTGGATTCGTAGCTGAA |
| *EMF1* Rev | CACCCACTTTCTCCTCAATAGC |
| *FT* For | CATCGTGTCGTGTTTATATTGTTTCG |
| *FT* Rev | CCTCCGCAGCCACTCTCC |
| *SEP1* For | ATGCTTGACCAGCTCTCGGATCTT |
| *SEP1* Rev | TGAGCTTGATGATGCGCGTAGGTA |
| *SEP3* For | ACGCCTTACAGAGAACCCAAAGGA |
| *SEP3* Rev | TTTGTCTCAGTCAGCATGCGTTCC |
| *ACTIN4* For | TCACCACAACTGCTGAACGTGA |
| *ACTIN4* Rev | TGCTTTCGCAATCCACATCTGC |
| *GAPC* For | TTGAAGGGTGGTGCCAAGA |
| *GAPC* Rev | CTTCGGATTCCTCCTTGATAGC |
| **Quantitative real time RT-PCR primers** | |
| *AP1* For | GCAGCACCAAATCCAGCATCC |
| *AP1* Rev | AGTTGTAAACGGGTTCAAGAGTCAG |
| *AP2* For | ATCCTACTACTCCACAAGATCACAAC |
| *AP2* Rev | AGAATCCGCCTCCGCTACC |
| *AP3* For GCCTCTGACATCATTACCTTCCATC | |
| *AP3* Rev | ACACACAATAAGCCTAAGCACTACTG |
| *PI* For | ACCAATGCTCCTCTTCTTGTTCTTC |
| *PI* Rev | ACTCTGTTGTTTGCGTTCTCTATCC |
| *AG* For | CGAGTATAAGTCTAATGCCAGGAG |
| *AG* Rev | GAGTAATGGTGATTGTTAGGTTGC |
| *SEP3* For | CGGTCGTCATCATCATCAACAACAAC |
| *SEP3* Rev | GGTCCTGCTCCCATTCCATCTTG |
